# Unlocking complex soil systems as carbon sinks: multi-pool management as the key

**DOI:** 10.1038/s41467-023-38700-5

**Published:** 2023-06-15

**Authors:** Gerrit Angst, Kevin E. Mueller, Michael J. Castellano, Cordula Vogel, Martin Wiesmeier, Carsten W. Mueller

**Affiliations:** 1 German Centre for Integrative Biodiversity Research (iDiv) Halle-Jena-Leipzig, Leipzig, Germany; 2 Institute of Biology, Leipzig University, Leipzig, Germany; 3 Biology Centre of the Czech Academy of Sciences, Institute of Soil Biology & Biogeochemistry, České Budějovice, Czech Republic; 4 Department of Biological, Geological and Environmental Sciences, Cleveland State University, Cleveland, OH USA; 5 Department of Agronomy, Iowa State University, Ames, IA USA; 6 Soil Resources and Land Use, Institute of Soil Science and Site Ecology, TU Dresden, Dresden, Germany; 7 Institute for Organic Farming, Soil and Resource Management, Bavarian State Research Center for Agriculture, 85354 Freising, Germany; 8 Chair of Soil Science, TUM School of Life Sciences Weihenstephan, Technical University of Munich, 85354 Freising, Germany; 9 Department of Geosciences and Natural Resource Management, University of Copenhagen, Copenhagen, Denmark

**Keywords:** Carbon cycle, Ecosystem ecology, Carbon cycle

## Abstract

Much research focuses on increasing carbon storage in mineral-associated organic matter (MAOM), in which carbon may persist for centuries to millennia. However, MAOM-targeted management is insufficient because the formation pathways of persistent soil organic matter are diverse and vary with environmental conditions. Effective management must also consider particulate organic matter (POM). In many soils, there is potential for enlarging POM pools, POM can persist over long time scales, and POM can be a direct precursor of MAOM. We present a framework for context-dependent management strategies that recognizes soils as complex systems in which environmental conditions constrain POM and MAOM formation.

## Abstract (web summary)

Increasing C storage in mineral-associated organic matter is insufficient due to diverse, environmentally specific persistent soil organic matter formation. Context-dependent management strategies highlighting the importance of particulate organic matter are necessary.

## Introduction

Since the late 1980s^1–3^, many studies have stated the necessity to distinguish particulate organic matter (POM) from mineral-associated organic matter (MAOM) in order to better understand soil organic matter (SOM) dynamics and manage soils as a carbon (C) sink^4^. The logic is simple: although SOM includes diverse biomolecules that are positioned continuously along many biophysical and spatiotemporal gradients in soil^5^, POM and MAOM are two pools that are reasonable to separate (physically) and differ broadly in their ecological functioning, chemical composition, and turnover times^6^. Particulate OM is mostly derived from partly decomposed plant fragments^6^, and where it is not occluded within aggregates^7^, it has a relatively short residence time^8^ and can be easily decomposed under the right environmental conditions. Contrastingly, MAOM is tightly bound to minerals or occluded within small microaggregates (<50 µm^9^) and assumed to persist in soil for hundreds to thousands of years^10^, even though MAOM can be recycled on shorter timescales^11^ and is a potential nutrient pool for plants^12^. The lower bioavailability of MAOM and its large contribution to bulk soil C storage in many soils has stimulated many researchers to mainly focus on factors that influence its formation, chemical composition, and accumulation.

Recently, much SOM research and conceptual development of soil management strategies has emphasized the remains of microbiota (or microbial necromass)^13–15^, which can constitute a substantial portion of MAOM^16^. To build-up microbial biomass, and microbial necromass retained in MAOM, some authors suggest manipulating plant inputs, e.g., by introducing plants that supply types of organic substrates that microorganisms can more efficiently convert to microbial biomass^13,14,17^ and thus enhancing microbial necromass. However, the performance of such microbe-and-MAOM-centric strategies for research and soil management may suffer from persistent uncertainty about the composition of MAOM and the efficiency of its formation from different precursors. For example, in many soils, biotic and abiotic factors likely allow plant-derived biomolecules to account for a substantial fraction of MAOM^16^. Particulate OM itself can be a precursor to MAOM, but the importance of such a link between these two pools is likely sensitive to environmental constraints. Recent studies of agricultural soil microcosms suggest that formation of MAOM is unrelated to POM, but linked to inputs of dissolved compounds leached from plant litter^18,19^, which can easily sorb to mineral surfaces (“direct sorption”) or be metabolized by the microbial community. Yet, similar elemental, isotopic, and chemical characteristics among POM and MAOM across diverse ecosystems^20–23^ indicate intricate links between MAOM formation and microbial depolymerization and transformation of POM into simpler forms and microbial necromass. Thus, different SOM precursors and formation pathways appear to have varying importance in different contexts^24^, and generalizations about MAOM formation mechanisms seem problematic. Dissolved organic compounds may be less relevant in systems that lack a thick organic layer or in which plant biomass or litter is regularly removed, such as in certain croplands^25,26^. Decomposing POM can also be a direct source of dissolved organic compounds in mineral soils and thus link POM and MAOM^22,27^. Likewise, microbial depolymerization and transformation of litter, or POM, and the related build-up of MAOM may be more or less efficient depending on multiple environmental constraints that determine microbial proliferation and the stabilization of microbial remains. For example, “recalcitrant” POM (with high lignin to nitrogen (N) or C:N ratios) can hamper formation of MAOM as compared to “high-quality” POM (low lignin:N or C:N ratios^28^).

Focusing research or management exclusively or primarily on MAOM also obscures several key facts: (i) C stocks in organic horizons and in POM within mineral soils can be large, and persist for hundreds to thousands of years (even if the residence time of each biomolecule or C atom is not long), (ii) some types of POM, such as that occluded in aggregates, are relatively stable as indicated by residence times of hundreds of years^29^), and (iii) proportionally, POM-C may contribute as much or more to total C storage than MAOM-C, such as in soils with a limited capacity for mineral protection of SOM^30,31^ or grassland soils in alpine or semi-arid environments^29,30^. In our view, emphasis toward MAOM also coincides with a problematic reduction in the number of studies and management strategies that consider the organic horizon, which is primarily comprised of POM and is a large pool of C and nutrients in many ecosystems. While the importance of POM has certainly been recognized for some soil systems and in some conceptual and quantitative models^4,32,33^, we see a critical need to develop a more holistic and integrative view of POM and MAOM dynamics, including their interactions and sensitivity to management practices and environmental conditions that vary in space and time.

In summary, we propose that understanding of SOM dynamics and effective management for C sequestration are hindered by an over-emphasis on MAOM, inattention to interactions between MAOM and POM, and context-blind generalizations about SOM dynamics. In our view, a recalibration of SOM research to address these hindrances will result in more integrative conceptual and quantitative models of SOM dynamics, enabling more accurate and applicable knowledge of the sources of SOM (i.e., microbial *versus* plant-derived organic matter^16,28,34^) and the functions and locations of SOM (in organic versus mineral horizons, in top- versus subsoils, in POM versus MAOM fractions).

### Developing a systems approach for SOM research and management

**Fig. 1:**
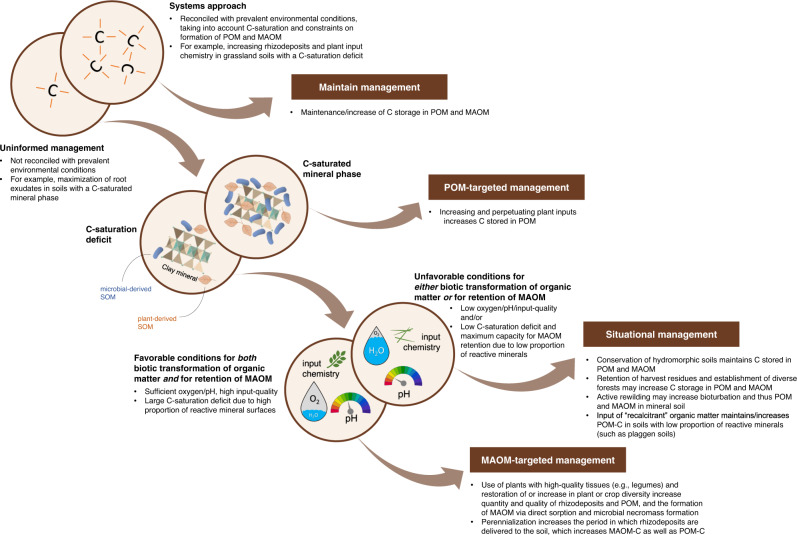
Systems approach for the contextualization of carbon-focused management strategies. Management strategies that are adapted to prevailing environmental conditions (i.e., following the systems approach) should be maintained, while strategies not well-adapted to site-specific conditions (i.e., uninformed) can be improved (first double circle from the upper left). The optimal management strategy depends on whether the soil has a carbon (C)-saturated mineral phase (second double circle) and favorable conditions for biotic transformation of organic matter and retention of mineral-associated organic matter (MAOM; third double circle). Recommended management strategies are indicated in boxes and examples provided in the bullet points below. POM particulate organic matter, SOM soil organic matter. Some elements in this figure adapted from Angst et al.^58^.

We advocate for a systems approach (Fig. 1) to studying and managing SOM that recognizes soils as complex systems in which POM and MAOM are distinctly important, but intertwined parts, and POM and MAOM formation, stocks, and stability determined by processes whose importance and outcomes are dependent on environmental factors and management practices that vary from site to site. Below, we outline this systems approach for maintaining and/or establishing soils as a C sink (Fig. 1). We specifically highlight how various constraints can affect the formation and interaction of MAOM and POM, with an emphasis on how optimal soil management might depend on C saturation, land use, or soil type.

### Targeting POM in systems with C-saturated mineral phase

We argue that whether C-focused management strategies should target POM or MAOM (or both) is principally determined by the C saturation of a soil. The C-saturation concept is based on the assumption that the amount of silt- and clay-sized minerals determine the overall capacity of a soil to store C^35–37^. The concept primarily focuses on MAOM as the major soil C pool and on the specific mineral surface area of fine-sized minerals as the main driver of MAOM stabilization. While MAOM-C indeed tends to “saturate” with increasing C contents, the capacity of a soil to store additional C is not reached at that point. Near and above the C-saturation threshold of a soil, formation of MAOM will be less efficient^13,18,38^, but not zero^39–41^. Likewise, there is ample evidence that additional (plant) inputs can continue to accumulate as labile (free) or stabilized (occluded) POM^42–47^. Thus, for soils (or soil horizons^48,49^) that are at or near their theoretical C-saturation limit, MAOM-centric strategies aimed at increasing C storage will be ineffective^48^ and may even be counterproductive^50,51^. For example, in C-saturated soils, promoting plants with ‘high quality’ litter or greater root exudation could diminish both POM pools (because of more complete decomposition of plant litter and mineralization of plant-derived C^28^) or MAOM pools (due to exudation-induced priming^50^). In our opinion, for soils with a C-saturated mineral phase, focus should rather be on increases in POM, e.g., via higher amounts of structural, perhaps more recalcitrant, plant inputs (Fig. 1). If these inputs are perpetuated, C storage in such soils can be increased in the long term via the rather labile POM pool, even if MAOM formation is low. For example, in forests managed for timber production, management practices that retain larger fractions of biomass residues (e.g., leaves and branches) could increase SOC stocks^52,53^, even if MAOM-C pools are close to saturation. In croplands, increased litter inputs combined with practices such as no or reduced tillage in agricultural systems may also increase the formation of aggregates^54–56^ and thus the persistence of POM occluded within these aggregates.

### Increasing MAOM relies on favorable soil conditions for biotic transformation of organic matter and for retention of MAOM

Globally, the average C-saturation deficit of surface soils (≤30 cm depth) is estimated to be roughly at 50%, and soils further from C-saturation may accrue C more effectively^48^. For these soils, improved management could increase MAOM-C. However, whether MAOM-targeted management in such soils is effective primarily depends on both (i) environmental conditions such as pH, chemistry of organic matter inputs, or availability of oxygen, and (ii) the overall proportion of reactive mineral surfaces. These parameters determine the rates at which soil fauna and microorganisms depolymerize and transform organic matter into simpler forms and microbial necromass, which is central to the formation of MAOM^14,20,23,57,58^, and the capacity of a soil to store C as MAOM, respectively. We thus expect that formation of MAOM can be boosted by implementation of appropriate management in soils with conditions that favor transformation of organic matter (e.g., organic matter inputs with low C/N and lignin/N or sufficient oxygen) *and* that have abundant reactive minerals, such as soils rich in silt and clay (Fig. 1).

For example, aerobic grassland soils with a C-saturation deficit and high abundance of reactive minerals provide the potential to increase C stored in MAOM via increased root exudates^59^ and structural plant inputs^60^. Grasslands typically have a continuous input of plant material but no organic horizons^61,62^, low C:N ratios and low ratios of lignin to N in plants^63^, and near-neutral soil pH^64^, resulting in an efficient transformation of plant-derived organic matter into microbial products and subsequent formation of more persistent MAOM. Recent studies suggest that suitable management strategies, such as optimized grazing intensities, multitrophic rewilding, or restoration of plant diversity, can alter the quantity of rhizodeposits and the quantity and quality of POM in grasslands, boosting formation of MAOM-C (*and* POM as precursor pool) both via direct sorption of dissolved organic matter and biotic transformation of POM^60,65–68^.

Likewise, soils in an intermediate development stage on loess-rich parent materials, such as Chernozems, Cambisols, or Luvisols, are usually fertile, well-aerated, and have a high reactive mineral surface area^69^. These characteristics provide favorable conditions for microbial transformation and stabilization of SOM, as indicated by high amounts of microbial necromass in bulk soil and SOM fractions^16,70^. Many of these soils are under agricultural use and consequently low in POM due to biomass export via harvest and rapid decomposition of plant inputs in these systems^69,71,72^. Total SOM stocks in these circumstances may be increased by an improved management of plant inputs (e.g., use of high-quality cover crops [legumes], cultivars with higher or deeper root-derived inputs, and/or perennials; retention of crop residues) in combination with reduced tillage, optimized fertilization, or organic amendments^56,68,73–75^ (but also refer to Schlesinger^76^). These management practices may eventually boost MAOM-C in the medium- to long-term via root exudates and plant-derived POM as precursor pools^48,59,77^.

### Reconsidering POM to meet specific soil and management conditions

Soils with adverse conditions for faunal and microbial activity have often hampered decomposition of plant residues that leads to the accumulation of POM both in mineral soils and in organic horizons, which can comprise high C stocks^78^. The conditions that favor POM accumulation (vs. MAOM formation) are likely caused by the interplay of various environmental factors (such as precipitation, topographic position, soil type, or vegetation)^32^ that cannot easily be shifted towards a state favorable for MAOM formation. Moreover, soils with low mineral surface area have a reduced capacity for retaining MAOM, so even if those soils have a C-saturation deficit and favorable conditions for biotic transformation of organic matter inputs, POM could be an important pathway for additional C sequestration. We argue that effective management of such soils as a C sink has to be situational and will require a renewed consideration of POM, especially where its formation and stabilization are more favorable (e.g., forests compared to most grasslands and some croplands; cold versus warm climates; acidic versus neutral soils; sandy soils; see below; Fig. 1).

For example, accumulation of POM and reduced formation of MAOM are often related to low-quality plant inputs (e.g., high C to N or lignin to N ratios), low soil N availability, and low soil pH values. Such conditions are often found in coniferous forests^69,79,80^ and beneath some deciduous hardwood stands or species^81^. Plant inputs with high C:N ratios, rich in tannins, waxes, and lignin, can hamper microbial metabolization of POM derived from these plant tissues and thus the efficiency with which MAOM is formed^82,83^. The typically low soil pH under trees with low-quality tissues^84,85^ can have further detrimental effects on the microbial and faunal community (e.g., absence of earthworms and inhibition of bacteria), reduce the number of mineral surfaces available for the sorption of organic matter^86^, and favor the dissolution of minerals (typically in Podzols). Trees associated with ectomycorrhizal fungi may be more likely to create conditions unfavorable for efficient bacterial conversion of plant litter and POM to MAOM^87–91^. In systems with one or more of these ‘unfavorable’ conditions, formation of MAOM may proceed relatively slowly^71^ and be limited to, or dominated by, direct sorption of dissolved organic compounds^92^. These conditions result in the formation of a thick organic layer, a high contribution of POM to total SOM pools^71^, and an elevated contribution of plant compounds to MAOM (and reduced contribution of microbial necromass) as compared to other ecosystems^16^.

Notably, an overly MAOM-centric view of soil C sequestration and ecosystem management may undervalue forests with large organic horizons and higher contributions of POM to SOM in mineral soils^71,78^. Implementing MAOM-centric management practices in such forests without consideration of POM may result in little change in total SOM stocks and net C sequestration^51^. We argue that in many forests, soil C stocks may be maximized by maintaining or increasing the diversity of overstory tree species^93^, including species with both high and low litter quality, which may allow for relatively large pools of POM *and* MAOM at the ecosystem scale^94^. Such interventions will be most effective when combined with management strategies that minimize the export of organic matter, *e.g*., through retention of harvest residues^52,95^ or active rewilding (Fig. 1). For example, retention of harvest residues in a eucalyptus plantation for a period of three years measurably increased both POM- and MAOM-C (2.1- and 1.2-fold)^96^. Moreover, re-establishment of populations of large animals or introduction of earthworms may increase bioturbation rates, with potential positive effects on MAOM-C^58,66,97^, negative effects on C stored in the forest floor, but no substantial changes in combined C stocks of forest floors and mineral soil^98^.

Similarly, hydromorphic soils have reduced oxygen availability and anoxic zones within the soil profile that lead to low redox potentials^99^ and the dominance of K-strategists^100^. This decelerates the accrual of microbial necromass in MAOM^101^ and favors the accumulation of POM, which accounts for up to 100% of C in Histosols and a major proportion of C in Cryosols^102–104^, paddy soils^105^, and Gleysols^99^. Particulate OM in cryogenic and hydromorphic soils could also play an important role in their response to climate change. Thawing permafrost may accelerate mineralization of POM (and loss of C) previously protected from decomposition via water logging or low temperatures^104^. Likewise, in areas where precipitation and anoxia is increasing, POM may accumulate (with potential decreases in MAOM^106,107^). Conversely, if hydromorphic soils are subject to reduced precipitation and increased aeration, losses of unprotected POM may contribute to reduced SOM stocks and substantial CO_2_ emissions^108,109^, unless MAOM forms at the same rate as POM is lost. Conservation and restoration of wetlands^110^ and peatlands thus appear to be the most reasonable interventions to maintain and increase storage of POM-C in soil systems that naturally preserve large amounts of rather labile C over millennia (Fig. 1).

We further argue that soils with a low proportion of reactive minerals surfaces, even if not C-saturated, are inappropriate targets for MAOM-centric management. Increases in MAOM-C in such soils will have little effect on the total SOC stock because their capacity to accrue C in MAOM is strongly limited and they are commonly dominated by C stored in POM^111^. Heathland or plaggen soils in north-western Europe^45,112^, for example, are mostly sand-rich, the C content is uncorrelated to MAOM^45,113^, and they store exceptionally high C amounts in POM^113,114^. This POM likely persists due to high contents of lipids, aliphatic compounds, and sterols that mainly originate from heathland vegetation and sustained high inputs of organic matter^112,113^. In our view, maintenance of and/or increase in C stocks in such sandy soils should be based on sustained inputs of “recalcitrant” organic matter with high C:N ratios, such as biochar^115^, to maintain and increase POM-C, rather than on a MAOM-focused approach using high-quality plant inputs^45^ (Fig. 1).

### Implications and outlook

We highlight that C sequestration in soil should not be a single-pool endeavor, and support our view by clarifying the varying relevance of POM and MAOM formation pathways across different environmental contexts, including levels of soil C saturation, climate, land use/cover, and soil type. Targeting MAOM alone will not optimize soils as a C sink in many contexts. To make full use of the C sequestration potential of soils, management strategies should recognize soils as complex systems and be tailored to the respective environmental conditions (Fig. 1). We advocate for the reconsideration of POM as a quantitatively and functionally important C pool and management target in a multitude of ecosystems. For example, POM is particularly important in ecosystems with a C-saturated mineral phase, with unfavorable conditions for biotic activity (and thus formation of MAOM), or with a low proportion of reactive minerals (such as in sand-rich soils) to accrue significant amounts of MAOM-C. Increasing and perpetuating organic matter inputs in such ecosystems is crucial to build and then maintain stocks of POM with a short mean-residence time compared to MAOM.

We further suggest that for many soils, C-sequestration can only be maximized by broadening the focus of MAOM-focused management strategies to build POM-C along with MAOM-C. Under suitable conditions, such as in fertile grassland soils with a C-saturation deficit and sufficient proportion of minerals, this could, for example, involve maintenance of diverse plant species with variable tissue quality (likely boosting POM- and MAOM-C^116^), instead of only promoting plant species with high-quality tissues (mainly boosting MAOM-C). In this context, we specifically see the need to more strongly address subsoils, whose large volume and C-saturation deficit enables additional storage of C in both POM and MAOM via rhizodeposits. We also believe a systems approach that aims to build both POM and MAOM is complementary to separate efforts to manage agricultural soils more holistically^117^, e.g., to boost soil health, biodiversity, nutrient-availability, and crop performance^118–121^.

A systems approach could also help develop sounder policies and standards for C-farming, which to date have not considered the fact that multiple C pools exist in soil^122^. More specifically, monitoring of C accrual (or maintenance) in both POM and MAOM is clearly needed to evaluate the “permanence” of soil C pools in response to C-farming schemes. We also advocate for allocating C-credits for accrual of C in POM, despite its potential lability, particularly in certain environmental contexts (Fig. 1); proper management could maintain large POM pools, like thick organic horizons, for decades to centuries, and even short-term C sinks entail measurable climate benefits^123^. We also believe that C-farming schemes will be more effectively implemented and subsequently improved by widespread and standardized assessment of POM and MAOM stocks, and environmental factors, as detailed in our systems approach^124^, both before and after implementation of C-farming strategies. Such data could, for example, reduce the risk of C loss and financial uncertainty for farmers, and help researchers test and improve newer models of SOM dynamics that explicitly incorporate POM and MAOM^33,125–128^ (and are thus more useful for informing soil management that is POM-and-MAOM-centric). To this end, fast and cost-effective approaches to quantify POM and MAOM, particularly simplified fractionation schemes^129^, are required for optimizing C-farming frameworks.

Collectively, we advocate for a more holistic view on SOM pools and their context-dependent formation and interactions; such a view will help to improve the efficiency of soil management for C sequestration and help recalibrate SOM research to be less narrowly focused on MAOM. Our systems approach (Fig. 1) enables the alignment of management strategies with the complexity of soils, which will be key to unlock and maintain them as a sustainable C sink.
